# Maternal bonding difficulties and postpartum depression in pediatric clinical practice: a longitudinal study in a Japanese cohort

**DOI:** 10.3389/fpsyt.2026.1939326

**Published:** 2026-09-28

**Authors:** Tomomi Yasaka, Takashi X. Fujisawa, Akemi Tomoda

**Affiliations:** 1 Division of Developmental Higher Brain Functions, United Graduate School of Child Development, Osaka University, Kanazawa University, Hamamatsu University School of Medicine, Chiba University, and University of Fukui, Osaka, Japan; 2 Saiseikai Fukuoka General Hospital, Fukuoka, Japan; 3 Fukuoka City Nanbu Developmental Support Center, Fukuoka, Japan; 4 Research Center for Child Mental Development, University of Fukui, Fukui, Japan; 5 Department of Child and Adolescent Psychological Medicine, University of Fukui Hospital, Fukui, Japan

**Keywords:** Edinburgh postnatal depression scale (EPDS), Japan, maternal bonding, mother-to-infant bonding scale (MIBS), perinatal mental health, postpartum depression, single-center cohort

## Abstract

**Background:**

Maternal bonding difficulties are an important yet under-recognized aspect of perinatal mental health, influencing both maternal well-being and child development. Although studies have examined postpartum depression and bonding impairment, few have investigated their longitudinal relationship within general pediatric settings in Japan.

**Methods:**

This single-center observational study analyzed data from 512 mother–infant dyads following delivery at a general hospital in Fukuoka, Japan. Maternal depressive symptoms and bonding were assessed at five days and one, two, and three months postpartum using the Edinburgh Postnatal Depression Scale (EPDS) and Mother-to-Infant Bonding Scale (MIBS), respectively. Latent growth models were estimated, and the resulting intercept and slope factor scores were subsequently subjected to Gaussian mixture-based clustering to identify trajectory-based subgroups. This analysis was conducted using an exploratory framework. The MIBS trajectory analyses were conducted on a subsample of 77 mothers with complete MIBS data, whereas the EPDS trajectory analyses used full information maximum likelihood-derived factor scores from 512 mothers.

**Results:**

In the linear mixed-effects model using all available paired observations, higher EPDS total scores were significantly associated with higher subsequent MIBS scores across the early postpartum period. Exploratory clustering of latent growth model-derived factor scores suggested three MIBS and four EPDS trajectory-based subgroups, characterized by different baseline levels and decreasing or persistent depressive symptom patterns. The EPDS total and subscale scores were also associated with MIBS scores. Because the MIBS trajectory analysis was based on a small, clinically selected, complete-case sample, subgroup-specific findings should be interpreted cautiously.

**Conclusions:**

Repeated EPDS and MIBS assessments in pediatric clinical practice may help characterize the associations between postpartum depressive symptoms and maternal bonding difficulties during the early postpartum period. Exploratory trajectory-based analyses suggested heterogeneous bonding-difficulty patterns, including a subgroup with increasing MIBS scores over the first three months postpartum. Further studies are required to determine whether such assessments can improve maternal and child outcomes.

## Introduction

The development of the mother–infant relationship is influenced by a complex interplay of multidimensional factors, including maternal and child characteristics and the caregiving environment. “Bonding” plays a particularly critical role in mother–infant interactions from birth (1, 2). The bonding process is regarded as unique and dynamic, continuing over an extended period, and is considered a crucial aspect of acquiring maternal identity (3). Bonding has been defined as a unidirectional emotional connection that a mother develops toward her child, emerging during the developmental period from birth to approximately one year of age. Proper bonding plays an important role in children’s socio-emotional development.

Conversely, a state of emotional rejection, characterized by maternal absence of positive feelings toward the infant, feelings of anger or rejection, or heightened anxiety and stress related to caregiving, has been described as “bonding disorder” (2, 4). Although not classified as an independent psychiatric diagnosis, impaired bonding is reportedly associated with externalizing behavioral problems at 18 months of age and an increased risk of childhood maltreatment (5). Thus, maternal bonding impairment is a significant clinical concern in perinatal mental health, as it is associated with impaired attachment, developmental difficulties in the child, and future risk of maltreatment. In Japan, early multidisciplinary intervention is recommended when bonding impairment is identified to support maternal bonding through regionally coordinated care systems (6).

Various maternal factors have been reported to be associated with bonding impairment, including unintended pregnancy and primiparity (7), negative attitudes toward pregnancy (8), unmarried status, elective cesarean section (9), and a history of childhood maltreatment (10). Pregnancy and childbirth are major life events that bring about substantial physical and psychological changes. Moreover, research shows that they can lead to mental health difficulties in women (1). Numerous studies have explored the relationship between maternal mental health difficulties and bonding impairment. These include associations with long-standing mental health issues originating in adolescence (11), anxiety (12, 13), post-traumatic stress disorder (10), and negative attachment styles toward the mother’s own caregiver (14, 15).

Among these, postpartum depression (PPD) is particularly critical. A recent Japanese study reported a 14.3% prevalence rate of PPD at one month postpartum (16). PPD can negatively impact child development, including difficulties in attachment formation, cognitive delays, and behavioral and emotional problems (17, 18). Numerous studies have also reported a significant association between PPD and impaired bonding (8, 11, 13, 19–22).

However, postpartum depressive symptoms and bonding impairment do not always coincide (23). Further, bonding impairment may persist even after depressive symptoms improve (23). While many mothers with depressive symptoms can still form normal relationships with their infants, some experience persistent difficulties in maternal emotional responses. This may adversely affect the formation of the mother–infant relationship over time (24). Furthermore, some studies suggest that the state of bonding in the early postpartum period is a stronger predictor of bonding quality at one year than depressive symptoms during the same period. Thus, the causal and temporal relationship between maternal mental health and bonding impairment remains insufficiently understood (22, 23).

Child-related factors associated with bonding impairment include female sex, the presence of medical conditions (25), sleep disturbances, preterm birth, and excessive infant crying (26). Environmental risk factors include intimate partner violence (12), lack of social support (21), low income, and low educational attainment (27). However, a systematic review of bonding impairment noted inconsistent findings across studies (27).

Meanwhile, screening instruments have been widely used in primary care to identify latent risks related to maternal mental health and mother–infant bonding (24), particularly the internationally recognized Edinburgh Postnatal Depression Scale (EPDS) and Mother-to-Infant Bonding Scale (MIBS). In Japan, the Ministry of Health, Labour and Welfare’s “Healthy Parents and Children 21” initiative, launched in 2006, set the reduction of PPD and child maltreatment-related deaths as key objectives. As part of this initiative, public health nurses utilized these scales in universal home visit programs (28).

Identifying and supporting mothers at risk of parenting difficulties, particularly from the perspective of perinatal mental health, is essential for promoting children’s well-being (29). Similarly, a biopsychosocial approach is essential in pediatric clinical settings to support caregivers and prevent child maltreatment. Multidisciplinary collaboration and a shared understanding among professionals involved in perinatal care are vital. From this perspective, we employed the EPDS and MIBS as part of our pediatric clinical practice.

Most studies on bonding impairment were conducted in obstetric or psychiatric settings and involved pregnant or postpartum women who provided consent and completed self-administered questionnaires longitudinally at home. However, to the best of our knowledge, no studies have focused on general pediatric outpatient settings. In Japan’s large-scale epidemiological study (Japan Environment and Children’s Study), only five core items from the MIBS were used, and full-scale assessments were not conducted (30).

Repeated assessment during the early postpartum period is clinically important because depressive symptoms and bonding difficulties may fluctuate during the transition from childbirth to early infant care. The selected time points corresponded to routine clinical contact points in our setting, including early postpartum hospitalization, the one-month infant health checkup, and pediatric follow-up visits up to three months postpartum. This longitudinal approach allowed us to explore heterogeneity in symptoms and bonding patterns beyond cross-sectional associations. This study examined the longitudinal associations between postpartum depressive symptoms and bonding difficulties by collecting full-item MIBS and EPDS data in a general pediatric outpatient setting where sustained postpartum contact with mothers is maintained.

## Methods

### Participants

This retrospective cohort study was conducted at a general hospital in Fukuoka City, an urban area in the Kyushu region of Japan. Data were collected from 603 mother–infant pairs who delivered at the hospital’s Department of Obstetrics and Gynecology between January 2013 and December 2018. Maternal psychological measures and demographic and socioeconomic data were obtained from obstetric and pediatric medical records, including intake records completed by social workers and assessments conducted by public health nurses. These data were collected as part of routine obstetric and pediatric clinical care and were not originally designed specifically for latent trajectory modeling. The collected background variables included maternal demographic characteristics, obstetric and psychiatric histories, family and socioeconomic factors, psychosocial support indicators, and infant characteristics. All information was anonymized and numerically coded to prevent personal identification. The exclusion criteria were as follows: non-Japanese nationality, multiple births, infants placed in foster care immediately after birth, cases in which the child was institutionalized within one month postpartum, and receipt of a one-month health checkup at another medical facility. Based on these criteria (**Figure 1**), 91 pairs were excluded. Subsequently, 512 mother–infant dyads were included in the main analytical sample. Repeated EPDS and MIBS assessments were performed as part of routine clinical care and pediatric follow-ups. Continued assessment was more likely when the mothers had psychosocial concerns, elevated EPDS or MIBS scores, or other clinical reasons for closer monitoring. Thus, the complete-case sample used for the MIBS trajectory-based subgrouping (n = 77) represented a clinically enriched follow-up group rather than a representative longitudinal subsample of the full cohort.

**Figure 1:**
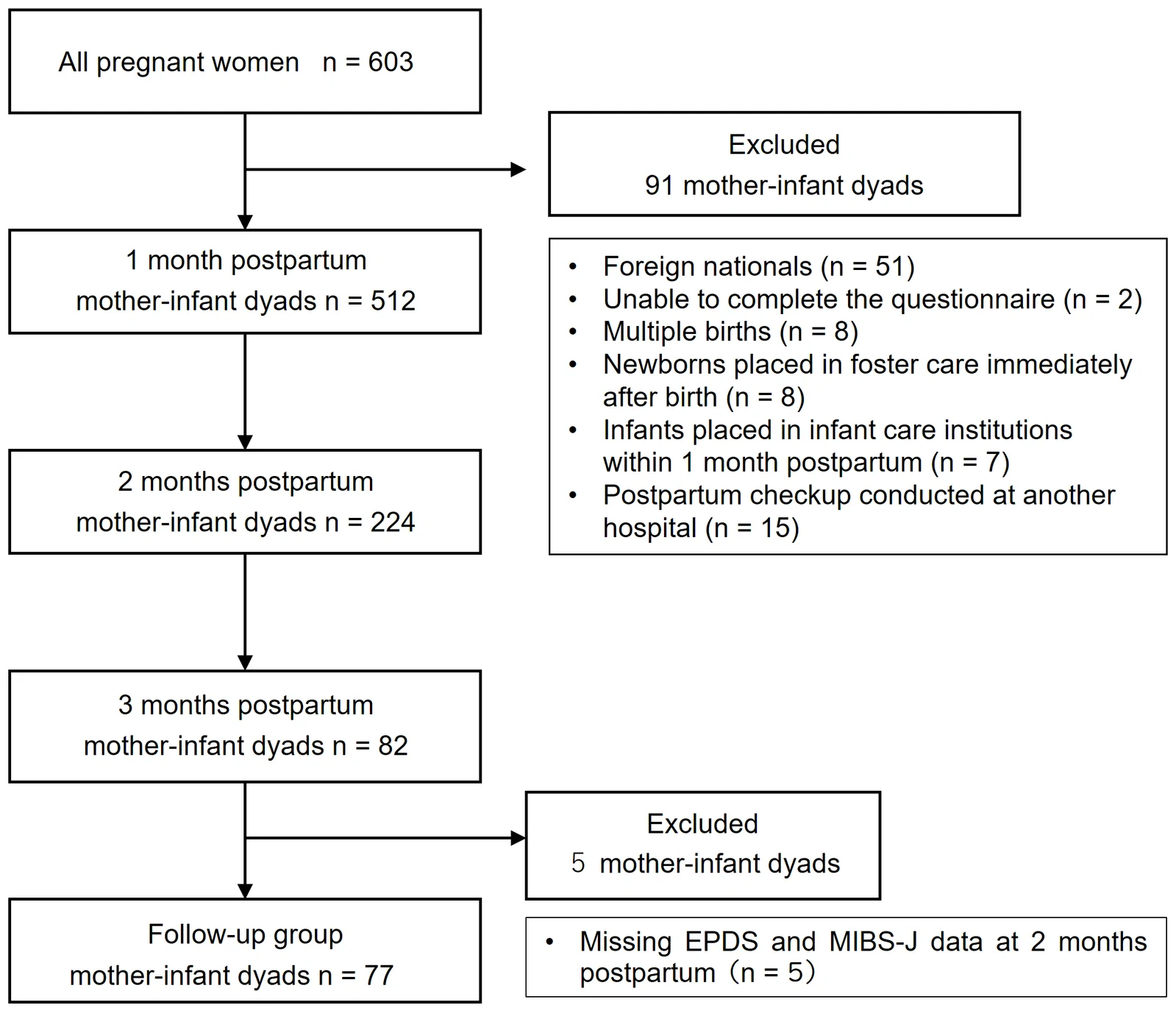
Flowchart of participant selection.

This study was approved by the Ethics Committee of Saiseikai Fukuoka General Hospital, Japan, on February 8, 2019. The committee did not issue an approval/reference number for this study. All procedures adhered to the Declaration of Helsinki and the Ethical Guidelines for Clinical Studies of the Ministry of Health, Labour and Welfare of Japan. Informed consent was obtained using the opt-out method; Information about the study was disclosed on the institutional website, and participants were given the opportunity to decline participation.

### Psychological measures

#### Mother-to-infant bonding scale

Maternal bonding with the infant was assessed using the MIBS, with the Japanese version used in this study (31, 32). Studies have demonstrated the reliability, validity, and clinical utility of the MIBS as a psychological measure (32). The Japanese version of the MIBS comprises 10 items rated on a four-point Likert scale, with total scores ranging from 0 to 30. However, item scores tend to be skewed toward lower values, resulting in a non-normal distribution of total scores (32). Higher total scores indicate greater difficulty in bond formation. For the MIBS cutoff, extant studies have proposed two thresholds: 2/3 (32–34) and 4/5 (35). Based on the similarity of participant characteristics to those in earlier studies, this study adopted the 2/3 cutoff.

Participants completed the assessments at three time points: one, two, and three months postpartum. The MIBS has been reported to comprise a two-factor structure: “anger and rejection” (items 2, 3, 5, 7) and “lack of affection” (items 1, 6, 8, and 10) (32, 36). Higher scores on the “anger and rejection” subscale have been associated with an increased risk of child maltreatment (35). However, the MIBS is a screening instrument for maternal bonding difficulties and does not assess child maltreatment. In this study, both the total and subscale scores were analyzed as primary outcomes, as evaluating both is essential for screening mothers who may require support (5).

#### Edinburgh postnatal depression scale

Maternal depressive symptoms were assessed using the EPDS, a widely used self-report questionnaire developed to screen for PPD (37). In Japan, the EPDS is commonly employed in clinical settings to screen for depressive symptoms. The reliability and validity of the Japanese version were established by Okano et al. (38), who recommended a cutoff score of 8/9.

Studies have suggested that administering the EPDS within a few days postpartum may be a useful screening tool for the early detection of postnatal depression (21, 39). Given that most mothers in Japan remain hospitalized for approximately five days after delivery, participants were asked to complete the EPDS on postpartum day five. Additional assessments were conducted at one, two, and three months postpartum, in line with the MIBS measurement time points.

In addition to depressive symptoms, the EPDS can help detect anxiety and other forms of psychopathology (40). A three-factor structure comprising anhedonia, anxiety, and depression has been reported (41, 42).

Given that maternal postpartum anxiety and depression have been associated with adverse developmental outcomes in infants (17, 18), this study analyzed both the EPDS total and subscale scores corresponding to the three factors as exploratory descriptive and correlational measures in relation to mother–infant bonding.

### Statistical analysis

Descriptive statistics were calculated for the demographic characteristics, total scores, and subscale scores of the MIBS and EPDS. Associations between demographic characteristics and EPDS and MIBS scores were examined using Spearman’s rank correlation coefficients. Subsequently, longitudinal associations between the MIBS and EPDS scores were investigated. Although studies have suggested that total MIBS scores tend to decline over time, increases in bonding difficulties are also observed, indicating potential heterogeneity in individual trajectories. The primary objective of the longitudinal analysis was to examine the association between EPDS and MIBS scores using all available paired observations. The secondary objective was to explore whether repeated assessments revealed heterogeneous patterns of depressive symptoms and bonding difficulties. For the principal longitudinal association analysis, we fitted a linear mixed-effects model with repeated MIBS total scores as the dependent variable. Because the EPDS was assessed at 0, 1, and 2 months postpartum, and the MIBS at 1, 2, and 3 months postpartum, EPDS scores were paired with subsequent MIBS scores at the next assessment period (EPDS 0 months with MIBS 1 month, EPDS 1 month with MIBS 2 months, and EPDS 2 months with MIBS 3 months). Time, EPDS total score, and the interaction between time and EPDS total score were included as fixed effects. Participant-level random intercepts were included to account for within-person correlations. The model used all available paired EPDS–MIBS observations. Participants contributed one to three observations depending on data availability, and observations with missing EPDS or MIBS values at the corresponding assessment point were excluded. Trajectory-based subgrouping was retained as a secondary exploratory, hypothesis-generating analysis.

To explore this heterogeneity, latent growth models (LGMs) were first estimated for the MIBS and EPDS scores across time. The models were fitted using full information maximum likelihood (FIML) in the lavaan package (version 0.6.20) in R (version 4.5.1). Individual-level factor scores for the intercepts and slopes were extracted from these models. These factor scores were subsequently subjected to Gaussian-mixture clustering using the mclust package (version 6.1.2) to identify subgroups with similar trajectory patterns. The same two-step LGM–mclust procedure was used for both MIBS and EPDS trajectory-based clustering; however, the analytical samples differed. MIBS clustering was based on 77 mothers with complete MIBS data at one, two, and three months postpartum. For EPDS, observed scores were available for 508, 512, and 223 mothers at five days, one month, and two months postpartum, respectively; the EPDS LGM was estimated using FIML. FIML-derived intercept and slope factor scores from 512 mothers were used for EPDS trajectory-based clustering. Thus, mclust was applied to the FIML-derived factor scores rather than directly to the incomplete repeated EPDS scores.

Candidate solutions with one to six components were compared using the VVV covariance structure, corresponding to ellipsoidal distributions with varying volume, shape, and orientation. In the mclust framework, the model with the largest Bayesian Information Criterion (BIC) is generally preferred. The solution with the largest BIC was selected as the primary model. Integrated completed likelihood (ICL), subgroup size, classification uncertainty, and clinical interpretability were additionally examined to assess the adequacy and stability of the selected exploratory subgroup solutions. Classification uncertainty was defined as one minus the maximum posterior classification probability, averaged across participants. This two-step approach differs from conventional growth mixture modeling, as growth parameters and subgroup membership were not simultaneously estimated. Accordingly, uncertainty in the individual growth parameters was not fully propagated into the clustering procedure. Further, uncertainty in subgroup assignment was not fully incorporated into the interpretation of subgroup membership. Therefore, the trajectory-based subgroup analysis was considered exploratory and hypothesis-generating.

Thus, the present clustering procedure should not be interpreted as a formal latent class model. Specifically, trajectory analyses for MIBS were conducted in a subsample of 77 participants with complete MIBS data across the one-, two-, and three-month postpartum assessments. Given the relatively small sample size, these analyses were considered exploratory, and the results should be interpreted with caution. To examine potential differences between participants included in the trajectory analysis (n = 77) and those without complete follow-up data (n = 435), group comparisons were conducted using chi-square tests, Mann–Whitney U tests, or independent t-tests on demographic characteristics and MIBS and EPDS scores.

## Results

### Demographic characteristics and follow-up status

**Table 1** presents the demographic and medical characteristics of all mother–infant dyads (N = 512), comparing those who completed the EPDS and MIBS at three months postpartum (follow-up group, n = 77) with those without follow-up data (non-follow-up group, n = 435). Compared with the non-follow-up group, mothers in the follow-up group were significantly more likely to be raising a child under three years of age (p = .014), to have experienced an unplanned pregnancy (p = .042), and to live in a stepfamily (p = .004). They also had a significantly higher prevalence of psychiatric history (p <.001) and current psychiatric treatment (p = .005).

**Table 1:** Demographic and medical characteristics of mother–infant dyads according to follow-up status (N = 512).

Variable	All participants (n=512)	Non-follow-up (n=435)	Follow-up (n=77)	Statistics	p-value
Maternal age at birth (years), mean (SD)	30.4 ± 6.8	30.2 ± 6.6	31.2 ± 7.6	t(97.4) = 1.07	0.287
Young pregnant woman (≤ 19years), n (%)	42 (8.2)	34 (7.8)	8 (10.4)	—	0.497^b^
Primiparity, n (%)	299 (58.4)	248 (57.0)	51 (66.2)	χ²(1) = 2.29	0.130^a^
Raising children under the age of 3, n (%)	131 (25.6)	120 (27.6)*	11 (14.3)*	χ²(1) = 6.09	0.014^a^
Cesarean, n (%)	166 (32.4)	138 (31.7)	28 (36.4)	χ²(1) = 0.64	0.423^a^
Single (not married), n (%)	130 (25.4)	111 (25.5)	19 (24.7)	χ²(1) = 0.02	0.876^a^
Unintended pregnancy, n (%)	221 (43.9)	180 (42.1)*	41 (54.7)*	χ²(1) = 4.12	0.042^a^
Stepfamily, n (%)	30 (5.9)	20 (4.6)*	10 (13.0)*	χ²(1) = 8.34	0.004^a^
History of psychiatric illness, n (%)	89 (17.4)	64 (14.7)**	25 (32.5)**	χ²(1) = 14.4	<0.001^a^
Undergoing psychiatric treatment, n (%)	35 (6.8)	24 (5.5)*	11 (14.3)*	χ²(1)= 7.90	0.005^a^
Family on welfare, n (%)	153 (29.9)	128 (29.4)	25 (32.5)	χ²(1) = 0.29	0.591^a^
Intimate partner violence, n (%)	36 (7.0)	28 (6.4)	8 (10.4)	—	0.225^b^
Public health nurse home visit refusal, n (%)	44 (9.2)	34 (8.4)	10 (14.1)	χ²(1) = 2.38	0.123^a^
Maternal adverse childhood experience, n (%)	140 (27.5)	113 (26.1)	27 (35.1)	χ²(1) = 2.64	0.104^a^
During the perinatal period					
Pregnancy complications, n (%)	88 (17.2)	67 (15.4)*	21 (27.3)*	χ²(1) = 6.48	0.011^a^
No return to parental home for childbirth, n (%)	350 (68.5)	289 (66.6)*	61 (79.2)*	χ²(1) = 4.83	0.028^a^
No family members to provide support, n (%)	51 (10.0)	45 (10.4)	6 (7.8)	—	0.679^b^
No partner (or husband) available for consultation, n (%)	133 (26.5)	114 (26.6)	19 (25.7)	—	1.000^b^
No person outside the family available for consultation, n (%)	14 (2.8)	11 (2.5)	3 (4.0)	—	0.446^b^
At one month postpartum					
No partner (or husband) available for consultation, n (%)	142 (27.7)	117 (26.9)	25 (32.5)	χ²(1) = 1.01	0.314^a^
No biological mother available for consultation, n (%)	158 (30.9)	124 (28.5)*	34 (44.2)*	χ²(1) = 7.51	0.006^a^
No non-family member available for consultation, n (%)	71 (13.9)	52 (12.0)*	19 (24.7)*	χ²(1) = 8.75	0.003^a^
Parenting is not enjoyable, n (%)	39 (7.7)	30 (6.9)	9 (11.8)	—	0.159^b^
Gestational age at birth (weeks), mean (SD)	38.6 ± 1.3	38.7 ± 1.3	38.5 ± 1.3	t(505) = 0.89	0.373
Female infant, n (%)	257 (50.2)	223 (51.3)	34 (44.2)	χ²(1) = 1.32	0.250^a^
Birth weight (g), mean (SD)	3004.7 ± 357.6	3016.0 ± 350.6	2940.9 ± 391.3	t(510) = 1.70	0.089
Low birth weight (<2,500 g), n (%)	39 (7.6)	27 (6.2)*	12 (15.6)*	χ²(1) = 8.18	0.004^a^

Comparisons are shown between mothers with complete follow-up data at 3 months postpartum (follow-up group, n = 77) and those without follow-up (non-follow-up group, n = 435). Values are presented as means ± SD or n (%). Percentages were calculated using non-missing observations as the denominator for each variable; therefore, the denominators may vary across variables because of missing data. ^a^ Pearson’s chi-square test; ^b^ Fisher’s exact test (two-sided), **<0.001, *<0.05.

During pregnancy, the follow-up group had a significantly higher proportion of pregnancy complications (p = .011) and was less likely to return to their parental home for childbirth (p = .028). At one month postpartum, a significantly higher proportion of the follow-up group reported being unable to consult with their cohabiting partner (p = .008), mother (p = .006), or anyone outside their family (p = .003).

Regarding infant characteristics, the follow-up group had a significantly higher proportion of low-birthweight infants (p = .004). No significant group differences were observed in terms of maternal age, primiparity, cesarean delivery, marital status, household receipt of public assistance, or gestational age.

### Mother-to-infant bonding scale and Edinburgh postnatal depression scale scores

The total MIBS scores at each time point showed a right-skewed, non-normal distribution, consistent with extant studies. **Table 2** compares the descriptive statistics for the MIBS and EPDS scores between the follow-up and non-follow-up groups. At one month postpartum, the mean MIBS total score was significantly higher in the follow-up group (mean ± SD, 2.2 ± 2.6) than in the non-follow-up group (mean ± SD, 1.2 ± 2.1, p <.001). Regarding subscales, “Anger and Rejection” scores were significantly higher in the follow-up group, whereas no significant differences were observed for “Lack of Affection.” Moreover, the proportion of mothers with MIBS total scores ≥ 3 was significantly higher in the follow-up group (29.9%) than in the non-follow-up group (12.6%, p <.001).

**Table 2:** Descriptive statistics of EPDS and MIBS scores.

Variable	All participants (n=512)	Non-follow-up (n = 435)	Follow-up (n = 77)	Statistic	p-value
EPDS 0m					
Total	4.6 ± 4.4	4.3 ± 4.4**	6.6 ± 4.3**	U = 22337	<0.001^a^
Anhedonia	0.2 ± 0.6	0.2 ± 0.7	0.3 ± 0.8	U = 12026	0.766^a^
Anxiety	1.3 ± 1.4	1.3 ± 1.4**	1.9 ± 1.6**	U = 20099	<0.001^a^
Depression	0.5 ± 1.0	0.5 ± 1.0**	0.9 ± 1.1**	U = 17402	<0.001^a^
EPDS 1m					
Total	3.8 ± 4.1	3.3 ± 3.7**	7.0 ± 5.0**	U = 25970	<0.001^a^
Anhedonia	0.9 ± 1.3	0.8 ± 1.2*	1.8 ± 1.5*	U = 11567	0.002^a^
Anxiety	0.2 ± 0.6	0.2 ± 0.5*	0.5 ± 1.1*	U = 10749	0.027^a^
Depression	0.4 ± 0.8	0.3 ± 0.8*	0.9 ± 1.2*	U = 11972	0.003^a^
MIBS 1m					
Total	1.2 ± 2.1	1.1 ± 2.0**	2.2 ± 2.6**	U = 19530	<0.001^a^
Anger & Rejection	0.7 ± 1.0	0.7 ± 1.0**	1.2 ± 1.2**	U = 18453	<0.001^a^
Lack of Affection	0.4 ± 1.1	0.4 ± 1.0	0.7 ± 1.5	U = 14638	0.083^a^
≥ cutoff value (%)					
EPDS 0m Total	15.7	13.4**	28.9**	χ² (1) = 11.74	<0.001^b^
EPDS 1m Total	12.7	8.7**	35.1**	χ² (1) = 40.92	<0.001^b^
MIBS 1m Total	15.2	12.6**	29.9**	χ² (1) = 15.03	<0.001^b^

Values are means (± SD) for follow-up and non-follow-up groups. Percentages were calculated using the number of participants with available data for each assessment point. Cutoffs: EPDS ≥ 9 and MIBS ≥ 3. Group differences were tested with Mann–Whitney U tests and χ² tests. EPDS, Edinburgh Postnatal Depression Scale; MIBS, Mother-to-Infant Bonding Scale. **<0.001 *<0.05.

For the EPDS, the mean total score at five days postpartum was significantly higher in the follow-up group (mean ± SD, 6.6 ± 4.3) than in the non-follow-up group (mean ± SD, 4.3 ± 4.4, p <.001). This trend persisted at one month postpartum for both the total and subscale scores. Furthermore, the proportion of mothers scoring ≥ 9 on the EPDS was significantly higher in the follow-up group at both five days (28.9% vs. 13.4%) and one month postpartum (35.1% vs. 8.7%; both p <.001).

Overall, these comparisons indicate that the follow-up group was clinically enriched and should not be regarded as a randomly selected longitudinal subsample of the full cohort.

**Table 3** shows the Spearman’s rank correlation coefficients between the MIBS and EPDS scores at one, two, and three months postpartum, with EPDS assessed at zero months. The variables included the total and subscale scores for both scales at each time point. Correlation coefficients were considered significant at p <.05 or p <.001. The EPDS total score at 0 months was significantly associated with the MIBS total and subscale scores at 1–3 months (r = .19–.39, p <.05). Concurrent correlations between the EPDS and MIBS scores at 1–3 months were stronger (r = .46–.63, p <.01). Notably, anhedonia was strongly associated with MIBS “Lack of Affection,” while anxiety was strongly associated with “Anger and Rejection.” These findings suggest that postpartum depressive symptoms are associated with bonding difficulties during the early postpartum period, with stronger concurrent associations at later time points. Further, specific depressive symptom dimensions may have different associations with bonding.

**Table 3:** EPDS and MIBS Spearman correlations (total and subscales) at 0–3 months postpartum.

Variable		MIBS								
		1 month			2 month			3 month		
		Total	Anger and rejection	Lack of affection	Total	Anger and rejection	Lack of affection	Total	Anger and rejection	Lack of affection
0m	Total	.385**	.385**	.241**	.363**	.328**	.268**	.311**	.324**	
	Anhedonia	.211**	.192**	.166**	.192**	.157**	.160**	.244**	.246**	
	Anxiety	.219**	.238**	.133**	.213**	.214**		.275*	.271*	.233*
	Depression	.277**	.255**	.183**	.240**	.191**	.205**			
1m	Total	.590**	.501**	.437**	.441**	.399**	.308**	.396**	.423**	.228*
	Anhedonia	.458**	.413**	.333**	.354**	.341**	.212**	.312**	.346**	
	Anxiety	.379**	.294**	.384**	.392**	.323**	.432**	.379**	.334**	.314**
	Depression	.346**	.292**	.258**	.264**	.197**	.229**	.328**	.338**	
2m	Total	.414**	.318**	.386**	.526**	.466**	.424**	.579**	.609**	.375**
	Anhedonia	.390**	.274**	.419**	.408**	.359**	.383**	.504**	.486**	.468**
	Anxiety	.394**	.333**	.383**	.481**	.446**	.377**	.500**	.490**	.371**
	Depression	.331**	.270**	.258**	.363**	.324**	.281**	.396**	.465**	
3m	Total	.228**		.295**	.423**	.388**	.251*	.630**	.632**	.433**
	Anhedonia				.429**	.483**	.237*	.518**	.579**	.450**
	Anxiety			.246**	.391**	.435**		.500**	.519**	.321**
	Depression				.259*	.272*		.460**	.480**	.287**

EPDS scores include baseline (0 month). Correlations were considered significant at *p < .05 and **p < .001.

### Longitudinal association between mother-to-infant bonding scale and Edinburgh postnatal depression scale scores

A linear mixed-effects model was fitted using all available paired observations, in which the EPDS scores at one, two, and three months postpartum were paired with the MIBS scores at one, two, and three months postpartum. Of the 512 dyads in the analytical cohort, 509 had at least one paired EPDS–MIBS observation available for this model, contributing 807 observations. Higher EPDS total scores were significantly associated with higher MIBS total scores across the early postpartum period (β = 0.153, 95% CI 0.122–0.184, p <.001). The main effect of time was not significant (β = −0.063, 95% CI −0.230 to 0.103, p = .456); the EPDS-by-time interaction was also not significant (β = 0.008, 95% CI −0.027 to 0.043, p = .644). These findings suggest that earlier depressive symptoms were consistently associated with subsequent bonding difficulties across repeated assessments, while the strength of this association did not significantly differ by time.

### Mother-to-infant bonding scale and background factors

Spearman’s rank correlation coefficients between MIBS and background factors are shown in **Tables 4A** and **4B**. Having another child in institutional care and reporting “parenting is not enjoyable” at one month postpartum were consistently and positively associated with MIBS across all time points. Primiparity was significantly associated with higher MIBS total and both subscales at one and two months postpartum, although not with “Lack of Affection” at two months. Having another child under 3 years old was negatively correlated with MIBS total and “Anger and Rejection” at one month, and positively associated with “Lack of Affection” at three months.

**Table 4A:** Associations between MIBS total and subscale scores and baseline background factors at admission.

Variable	1 month			2 month			3 month		
	Total	Anger and rejection	Lack of affection	Total	Anger and rejection	Lack of affection	Total	Anger and rejection	Lack of affection
Young pregnant women (≤ 19 years)	0.020	0.036	-0.038	0.013	-0.005	0.071	0.053	0.067	0.054
Fertility treatment	0.041	0.031	0.043	0.069	0.018	0.130	-0.097	-0.116	-0.112
Primiparity	0.294**	0.336**	0.124**	0.161*	0.187**	0.044	-0.158	-0.126	-0.127
Raising children under the age of 3	-0.242**	-0.263**	-0.082	-0.019	-0.044	0.092	0.148	0.098	0.254*
Cesarean	-0.041	-0.048	0.041	-0.022	-0.044	-0.038	-0.150	-0.270*	0.038
Single (not married)	-0.142**	-0.114**	-0.130**	-0.162*	-0.112	-0.144*	-0.058	0.028	-0.149
Pregnancy before marriage	0.100*	0.086	0.033	-0.043	-0.058	-0.039	-0.171	-0.155	-0.124
Unplanned pregnancy	0.038	-0.006	0.029	-0.022	-0.043	0.002	-0.058	-0.037	-0.024
Stepfamily	-0.070	-0.067	-0.052	-0.044	-0.013	-0.095	-0.011	0.032	-0.144
Mother’s stepchild	-0.078	-0.081	-0.044	-0.068	-0.049	-0.08	-0.046	-0.023	-0.111
Father’s stepchild	0.042	0.073	-0.044	0.004	0.036	-0.064	0.080	0.137	-0.113
Pregnancy complications (other than mental disorders)	0.138**	0.138**	0.120**	0.117	0.128	0.046	-0.044	-0.086	0.005
History of psychiatric illness	0.077	0.069	0.081	0.089	0.086	0.050	0.123	0.172	-0.042
Undergoing psychiatric treatment	0.066	0.074	0.070	0.055	0.036	0.065	0.096	0.111	-0.030
Family on welfare	-0.105*	-0.123**	-0.039	-0.158*	-0.11	-0.073	-0.009	0.109	-0.170
Economic anxiety	0.046	0.033	0.001	0.001	-0.019	0.089	<0.001	0.064	-0.077
Intimate partner violence	-0.012	-0.025	0.013	0.179**	0.194**	0.157*	0.126	0.195	-0.019
Public health nurse home visit refusal	0.047	0.018	0.063	0.039	0.051	0.062	0.181	0.151	0.154
Mother’s own upbringing (parental divorce, abuse, etc.)	-0.085	-0.091*	-0.031	-0.061	-0.058	0.053	0.109	0.137	0.045
Abortion	0.008	-0.016	0.049	0.004	0.018	0.014	0.103	0.079	0.118
Miscarriage	-0.077	-0.066	-0.088*	-0.060	-0.016	-0.137*	0.018	0.077	-0.202
Other children in institutional care	0.144**	0.103*	0.163**	0.167*	0.173**	0.209**	0.273*	0.297**	0.305**

Assessed using Spearman’s rank correlation coefficients; correlations were considered significant at *p < .05 and **p < .001.Bold values indicate significant correlations.

**Table 4B:** Associations between MIBS total and subscale scores and perinatal and postpartum background factors.

Variable	1 month			2 month			3 month		
	Total	Anger and rejection	Lack of affection	Total	Anger and rejection	Lack of affection	Total	Anger and rejection	Lack of affection
During the perinatal period									
Death of a close person	0.051	0.061	0.057	0.143*	0.184**	0.064	0.104	0.159	-0.011
Not returning home to give birth	-0.048	-0.081	0.011	-0.019	-0.032	0.022	0.097	0.066	0.103
No support	-0.006	-0.003	<0.001	-0.058	-0.054	0.001	-0.041	-0.011	-0.092
No family members to provide support	-0.035	-0.021	-0.029	-0.047	-0.013	-0.026	0.050	0.109	-0.053
Unable to consult with husband	-0.082	-0.081	-0.081	-0.016	-0.025	0.018	0.078	0.154	-0.017
No partner (or husband) available for consultation	0.112*	0.078	0.098*	0.168*	0.127	0.162*	0.226*	0.268*	0.113
No non-family member available for consultation	0.042	0.039	0.072	0.082	0.110	0.092	0.015	0.048	0.005
Smoking	-0.018	-0.038	0.037	0.052	0.070	0.048	0.066	0.115	0.008
Alcohol	-0.025	-0.029	0.045	-0.018	-0.026	0.076	-0.094	-0.064	-0.113
At one month postpartum									
No childcare support	-0.134**	-0.122**	-0.090*	-0.044	-0.042	-0.043	-0.086	-0.033	-0.111
Unable to consult with husband	-0.035	-0.026	-0.039	-0.039	-0.042	0.018	0.012	0.056	-0.026
No partner (or husband) available for consultation	0.129**	0.083	0.123**	0.136*	0.078	0.187**	0.097	0.082	0.075
No biological mother available for consultation	0.029	0.033	0.023	-0.02	-0.042	0.066	0.196	0.175	0.109
No biological mother	0.052	0.043	0.057	0.013	-0.043	0.131	0.180	0.109	0.179
No non-family member available for consultation	0.135**	0.124**	0.109*	0.129	0.061	0.222**	0.141	0.113	0.165
Parenting is not enjoyable	0.378**	0.326**	0.452**	0.361**	0.345**	0.400**	0.370**	0.376**	0.349**
Infant									
Gestational age at birth (weeks)	0.117**	0.125**	0.066	0.028	0.029	0.009	0.009	0.062	-0.020
Female infant	-0.017	-0.009	-0.017	-0.160*	-0.142*	-0.103	-0.198	-0.178	-0.095
Birth weight	0.005	0.036	0.004	0.057	0.063	0.097	-0.118	-0.049	-0.170
Low birth weight (<2,500 g)	0.075	0.082	-0.009	0.003	0.017	-0.020	-0.014	-0.049	0.004
Formula only	0.056	0.050	0.049	0.039	0.031	-0.002	0.051	0.020	-0.016
Monthly body weight gain (g)	-0.065	-0.083	0.011	-0.021	-0.016	0.010	-0.110	-0.026	-0.140
Physical problems in infants	-0.002	0.013	-0.033	-0.030	-0.030	-0.043	0.002	0.041	-0.092

Assessed using Spearman’s rank correlation coefficients; correlations were considered significant at *p < .05 and **p < .001.Bold values indicate significant correlations.

The inability to consult with a partner during pregnancy was associated with higher MIBS total and “Lack of Affection” at one and two months postpartum, and with higher MIBS total and “Anger and Rejection” at three months. Further, the inability to consult with a partner after childbirth was associated with higher MIBS total and “Lack of Affection” scores at one and two months postpartum. Additionally, the inability to consult with anyone other than family was associated with higher MIBS total and both subscales at one month, and “Lack of Affection” at two months. Gestational age showed a weak positive correlation with MIBS total and “Anger and Rejection” at one month. Having a female infant was negatively correlated with MIBS total and “Anger and Rejection” at two months. Living in a household receiving public assistance was negatively correlated with MIBS total and “Anger and Rejection” at one and two months postpartum. In contrast, maternal psychiatric history and current psychiatric treatment were not significantly associated with MIBS scores.

### Longitudinal analysis of maternal bonding and depressive symptoms

An LGM was estimated using FIML to examine MIBS total scores collected at one, two, and three months postpartum. This model demonstrated excellent model fit (χ² = 0.724, df = 1, p = .395, CFI = 1.00, TLI = 1.01, RMSEA = 0.00, SRMR = 0.02). The estimated mean slope was not statistically significant (est = −0.228, p = .175), suggesting no overall change in bonding difficulties at the group level. However, significant variances in both the intercept (var = 7.32, p <.001) and slope (var = 2.34, p <.001) indicated substantial individual differences in the initial bonding levels and trajectories over time.

To explore this heterogeneity, individual-level intercept and slope factor scores derived from the LGM were subjected to Gaussian mixture clustering using mclust. Candidate solutions with one to six components were compared using the VVV covariance structure. The three-component solution had the largest BIC and was selected for exploratory description (BIC = −544.559; ICL = −553.267; **Supplementary Table 1**). As this subgrouping was based on the estimated LGM-derived factor scores in a small complete-case sample, the resulting subgroups should be interpreted as exploratory trajectory-based patterns. The identified subgroups were characterized as follows:

- Subgroup 1: Low-stable bonding difficulties, n = 41 (53.2%);
- Subgroup 2: High-increasing bonding difficulties, n = 22 (28.6%); and
- Subgroup 3: Moderate-decreasing bonding difficulties, n = 14 (18.2%).

Group-level trajectory patterns are illustrated in **Figure 2A**.

**Figure 2:**
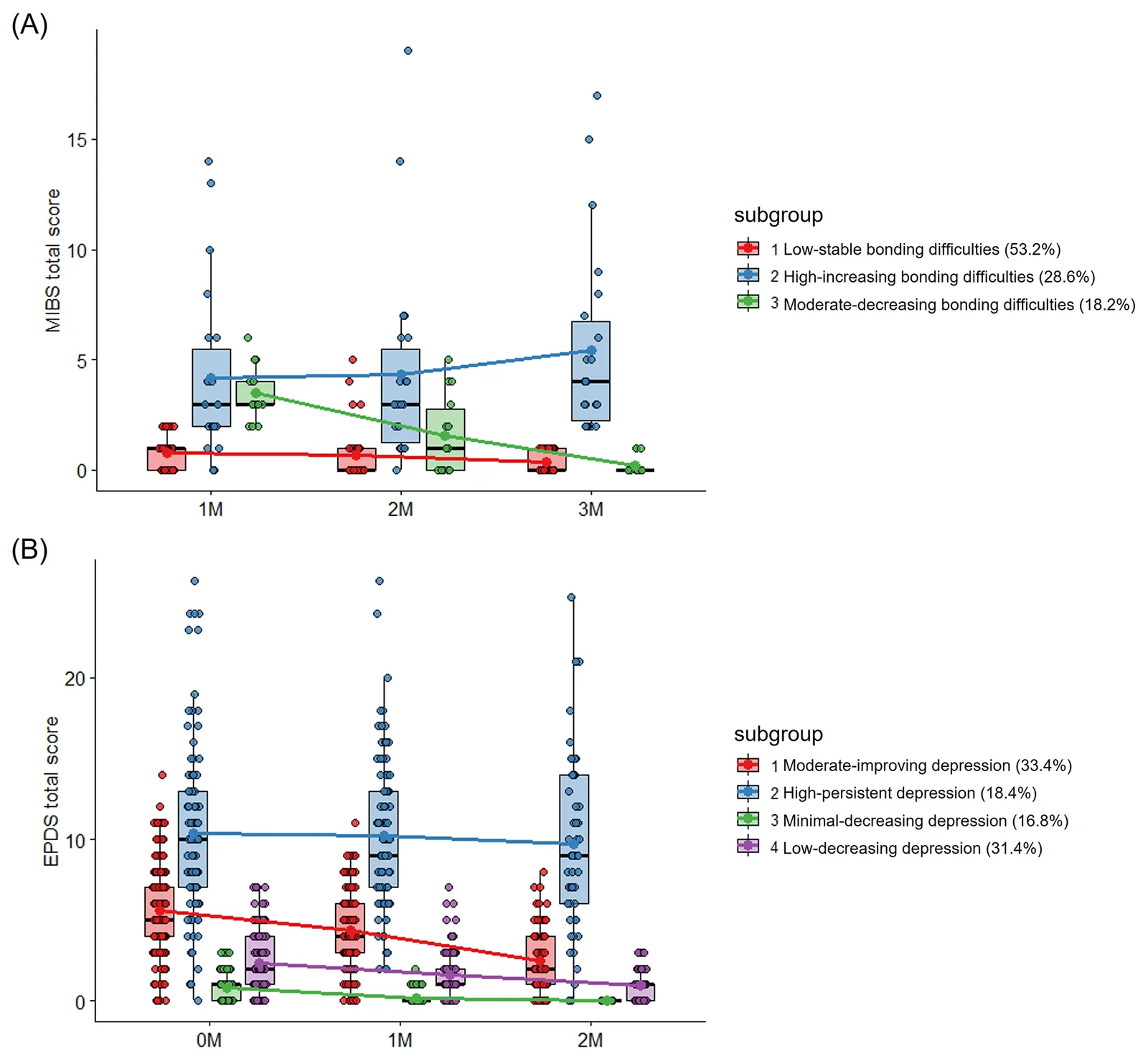
Exploratory trajectory-based subgroups derived from Gaussian mixture clustering of individual latent growth factor scores. **(A)** MIBS total score trajectories from one to three months postpartum. The three-component VVV solution had the largest BIC in mclust and included low-stable, high-increasing, and moderate-decreasing bonding-difficulty patterns. **(B)** EPDS total score trajectories from zero to two months postpartum. The four-component VVV solution had the largest BIC in mclust and reflected different baseline levels and decreasing or persistent depressive-symptom patterns.

Similarly, an LGM was estimated for the EPDS scores of the 512 mothers using FIML. The model demonstrated excellent fit (χ² = 0.06, df = 1, p = .80, CFI = 1.00, TLI = 1.01, RMSEA = 0.00, SRMR = 0.003). The estimated mean slope was significantly negative (est = −0.83, p <.001), indicating an overall decrease in depressive symptoms over time. Significant variances in both the intercept (var = 13.45, p <.001) and slope (var = 4.89, p <.001) indicated heterogeneity in individual trajectories.

Clustering based on the FIML-derived intercept and slope factor scores from the 512 mothers was conducted using mclust. Candidate solutions with one to six components were compared using the VVV covariance structure. The four-component solution had the largest BIC and was selected for exploratory description (BIC = −3751.202; ICL = −3890.019; **Supplementary Table 1**). These subgroups were characterized as follows:

- Subgroup 1: Moderate-improving depression, n = 171 (33.4%);
- Subgroup 2: High-persistent depression, n = 94 (18.4%);
- Subgroup 3: Minimal-decreasing depression, n = 86 (16.8%); and
- Subgroup 4: Low-decreasing depression, n = 161 (31.4%).

Group-level trajectory patterns are illustrated in **Figure 2B**.

Cross-tabulation analysis revealed a significant association between MIBS and EPDS subgroup membership (χ² (6) = 17.49, p = .008), with a Cramer’s V of.34 (95% CI [.25–.49]), indicating a moderate association between bonding and depressive symptom trajectories. Given the exploratory nature of the clustering approach, the two-step use of LGM-derived factor scores, the small complete-case sample for MIBS clustering, and classification uncertainty, these subgroup classifications should be interpreted with caution.

### Associations of depressive symptoms and background factors with maternal bonding

To examine the exploratory associations between earlier depressive symptoms and MIBS scores at three months postpartum, multiple regression analyses were conducted with MIBS total scores at three months postpartum as the dependent variable and EPDS total scores at 0–2 months and changes in EPDS scores during 0–1 and 1–2 months as predictors. The results indicated that the EPDS total score at 0 months and increases in EPDS scores during 0–1 and 1–2 months were significantly associated with higher MIBS total scores at 3 months (**Table 5**). Because earlier MIBS scores were not included as covariates, this analysis was interpreted as an exploratory association with three-month MIBS scores.

**Table 5:** Exploratory associations of early EPDS scores and EPDS score changes with three-month MIBS scores.

Predictor	B	SE	β	t	p-value	95% CI	Tolerance	VIF
(Constant)	-0.062	0.620	—	-0.101	0.920	[-1.297, 1.173]	—	—
EPDS total score (0 month)	0.320	0.084	0.422	3.812	<0.001	[0.153, 0.487]	0.791	1.265
ΔEPDS total score (0–1 month)	0.270	0.079	0.369	3.433	0.001	[0.113, 0.427]	0.837	1.195
ΔEPDS total score (1–2 month)	0.308	0.079	0.413	3.897	<0.001	[0.151, 0.465]	0.861	1.161

## Discussion

This study collected longitudinal data on maternal postpartum depressive symptoms (EPDS) and mother-to-infant bonding (MIBS) among mothers who delivered at a general hospital and examined their associations with each other and various background factors. A mixed-effects analysis using all available paired EPDS–MIBS observations showed that higher EPDS total scores were associated with higher subsequent MIBS total scores across the early postpartum period. This provides population-level evidence for an overall association between postpartum depressive symptoms and bonding difficulties. Trajectory-based clustering analysis was retained as a secondary exploratory approach to describe potential heterogeneity in longitudinal patterns, which would not be fully captured by average associations alone. In particular, the low-stable, high-increasing, and moderate-decreasing MIBS patterns suggest that bonding difficulties may follow different courses during the early postpartum period. Because these findings were based on an exploratory complete-case analysis, they should not be interpreted as definitive latent classes or evidence for distinct clinical subgroups. Rather, they suggest that repeated EPDS and MIBS assessments may help describe changes in psychological distress and bonding difficulties over time.

Latent growth modeling, followed by clustering of individual growth parameters, suggested three exploratory trajectory-based subgroups of MIBS and four exploratory trajectory-based subgroups of EPDS. The MIBS analysis identified low-stable, high-increasing, and moderate-decreasing bonding difficulty patterns. Meanwhile, the EPDS analysis reflected different baseline levels and decreasing or persistent depressive symptom patterns. These findings underscore the value of repeated rather than single-time assessments for describing the heterogeneity in postpartum depressive symptoms and bonding difficulties.

When comparing mothers who were followed up for at least three months with those who were not, the follow-up group showed significantly higher EPDS and MIBS total and subscale scores during the early postpartum period, and higher proportions exceeding clinical cutoffs. Possibly, the follow-up group included mothers at an elevated risk of mental health problems and bonding difficulties and may not represent the entire cohort. Still, this underscores the need for psychological support and tailored interventions. Studies have consistently emphasized the significance of addressing maternal perinatal mental health in promoting healthy development in children (17, 18, 29). Moreover, recent research has shown that early postpartum bonding difficulties are linked to atypical socio-emotional development in children (43, 44). Clinically, these findings suggest that pediatric practice may benefit from repeated rather than single-point EPDS and MIBS assessments. A single early postpartum score may not capture mothers whose bonding difficulties emerge, persist, or increase after the initial assessment. These exploratory findings may help clinicians recognize mothers who may benefit from closer monitoring, empathic consultation, and linkage to mental health or community support services when clinically indicated. These implications should be regarded as hypotheses for future prospective studies, rather than as evidence of intervention effectiveness.

Several correlations were found between MIBS scores and EPDS total and subscale scores, consistent with extant research (23). The MIBS subscale “Anger and Rejection,” which has been associated with increased risk of child maltreatment (5, 35, 45), was weakly correlated with EPDS “Anxiety” at five days postpartum and moderately correlated at one month postpartum. This is consistent with reports showing that postpartum anxiety and depressive symptoms are negatively associated with bonding (13), and that MIBS “Anger and Rejection” and “Lack of Affection” correlated with EPDS “Anxiety” and “Anhedonia,” respectively (19). Subscale-level examination of EPDS responses may provide additional clinical information beyond the total score, because anhedonia, anxiety, and depressive mood may relate differently to maternal bonding difficulties. In the present study, these subscale analyses were exploratory and should be interpreted as descriptive associations requiring replication. Notably, elevated MIBS scores do not indicate child maltreatment. Instead, they may reflect maternal emotional difficulties in bonding, which, together with other clinical and psychosocial information, can inform the need for support.

Among background factors, primiparity, non-psychiatric physical illness, inability to consult with a partner during pregnancy or postpartum, lack of someone to consult outside the family, and gestational age were weakly to moderately correlated with bonding difficulties. These results are consistent with reports linking bonding impairment to primiparity (7, 8, 46, 47) and limited social support (21, 48). Kanekasu et al. (48) reported that only postpartum social support predicted MIBS scores at three months, emphasizing the need to further examine the role of prenatal support. In the present study, the inability to consult with a partner during pregnancy was weakly associated with higher MIBS total scores at three months, suggesting that insufficient prenatal social support may be associated with greater postpartum bonding difficulties. Because our sample included a relatively high proportion of socially high-risk mothers, such as those with financial or relational difficulties, these associations may have been accentuated.

Meanwhile, unmarried status, smoking during pregnancy, young maternal age, and inadequate prenatal care were not significantly correlated with MIBS scores, contrary to studies that identified these variables as risk factors for child maltreatment. Similarly, receipt of public assistance, an indicator of economic hardship, was not associated with bonding difficulties, consistent with research suggesting that low income alone does not necessarily predict poor bonding (43). This may be because mothers who receive public assistance may already have access to certain forms of institutional support. Thus, the presence or absence of sustained social support during pregnancy may be more critical for bond formation than economic hardship itself (21).

Unintended pregnancy has been reported as a predictor of child maltreatment (49, 50) and a risk factor for bond impairment (7). Although the prevalence of unintended pregnancy was high overall and even higher in the follow-up group, it was not directly correlated with MIBS scores in this study. In contrast, having previously placed a child in institutional care and reporting that “parenting is not enjoyable” were consistently associated with higher MIBS total and subscale scores across time points. This suggests persistent bonding difficulties from the early postpartum period. As such, mothers with past parenting difficulties may experience recurrent bonding problems if adequate support is not provided, thereby increasing the risk of child maltreatment. Although no physical signs suggestive of abuse (e.g., failure to thrive, unexplained injuries, and shaken baby syndrome) were observed in the infants, the inability to consult with a partner or family members was positively associated with MIBS “Anger and Rejection” and “Lack of Affection.” These findings support studies linking emotional detachment and loss of enthusiasm for parenting with increased maltreatment risk (32) and highlight the importance of providing mothers with safe opportunities for consultation in pediatric clinical settings.

Although bonding difficulties generally improve as the child grows (51), exploratory trajectory-based analysis identified a subgroup whose MIBS scores increased over the first three months postpartum. Descriptive and supplementary exploratory analyses suggested that EPDS-related symptom dimensions, including anhedonia, may differ across MIBS trajectory-based patterns. However, because these analyses were based on a small complete-case sample and small subgroup sizes, these findings should not be interpreted as evidence that anhedonia predicts subgroup membership or as identifying a definitive clinical subgroup. Rather, they suggest that EPDS symptom dimensions warrant attention in future studies on PPD symptoms and bonding difficulties. Larger prospective studies are needed to clarify whether anhedonia or other EPDS symptom dimensions are associated with persistent or increasing MIBS scores.

The exploratory findings also highlight a potential gap between psychological distress and access to psychiatric care. Even among mothers with increasing bonding difficulties, current psychiatric treatment was not clearly overrepresented. This should be interpreted cautiously but is consistent with reports that awareness of and access to psychiatric care remain limited among postpartum mothers in Japan (52). A substantial proportion of mothers in the high-increasing MIBS subgroup also belonged to the EPDS subgroup characterized by persistently high depressive symptoms. This descriptive overlap suggests that depressive symptom burden and bonding difficulties may co-occur. However, it should be interpreted cautiously given the exploratory nature of the subgroup analysis. Studies have shown that unrecognized depression underlies anxiety-related bonding difficulties (53) and that interventions focusing on bonding are effective for mothers with PPD. Therefore, mothers exhibiting increasing MIBS scores but not yet engaged in psychiatric care may warrant closer clinical attention when psychological distress or bonding difficulties are also present. Together, these exploratory findings suggest that mothers with increasing MIBS scores, particularly those with elevated anhedonia, may require careful clinical attention.

Finally, this study has several limitations. First, although the subgroup solutions were selected using the correct mclust BIC convention, the trajectory-based subgroup analyses should be interpreted with caution. The dataset was collected as part of routine clinical care and was not originally designed for latent trajectory modeling. Clustering was performed on estimated LGM-derived factor scores rather than through the simultaneous estimation of growth parameters and class membership. Therefore, uncertainty in the estimated growth parameters was not fully propagated into the clustering step, while uncertainty in subgroup assignment was not fully incorporated into the interpretation of subgroup membership.

Classification uncertainty remains a concern, particularly for the small MIBS complete-case sample. The MIBS clustering analysis was based on only 77 mothers, and the smallest subgroup included 14 mothers, limiting the analysis of subgroup characteristics. Moreover, this complete-case sample was clinically enriched because continued follow-up was more likely among mothers with psychosocial concerns, elevated EPDS or MIBS scores, or other clinical reasons for closer monitoring. This indicates potential attrition or follow-up selection bias and limits the representativeness and generalizability of the identified trajectory-based subgroups. Therefore, the identified MIBS trajectory-based subgroups may reflect selective clinical follow-up rather than naturally occurring patterns in the overall cohort. Because the trajectory-based subgrouping was based on a small and clinically selected complete-case sample, these findings should be interpreted as exploratory and hypothesis-generating rather than definitive evidence for distinct latent classes in the broader postpartum population. Further, although alternative component solutions were examined using model-selection indices, uncertainty regarding the optimal number of subgroups remains. These exploratory subgroup solutions require validation in larger prospective cohorts.

Second, although the mixed-effects model used all available paired EPDS–MIBS observations, the analysis remained observational and could not establish temporal precedence or causality. In the present study, however, trajectory-based analysis was retained as an exploratory approach because our aim was to describe the relationship and correspondence between repeated EPDS and MIBS patterns, rather than to model average time effects.

Third, maternal attachment styles were not assessed. Fourth, the single-site design limits the generalizability of the results. Fifth, because the EPDS and MIBS measurements partially overlapped in time, the present analyses could not establish temporal precedence or causal relationships between depressive symptoms and bonding difficulties. Child maltreatment was not measured, and screening or referral interventions were not formally evaluated. Moreover, the assessments were conducted as part of clinical care, and some mothers received advice or referrals to local services. Therefore, the study included minor interventional elements and could not be considered purely observational. Accordingly, the findings should be interpreted as associations between questionnaire-based measures rather than as evidence that depressive symptoms cause bonding difficulties or that screening prevents maltreatment.

In conclusion, this study longitudinally examined the association between postpartum depressive symptoms and maternal bonding difficulties using the EPDS and MIBS in a single-center pediatric clinical cohort. EPDS total and subscale scores were associated with MIBS scores during the early postpartum period. Exploratory trajectory-based clustering suggested heterogeneous MIBS patterns, including a subgroup with increasing scores over the first three months postpartum. These findings suggest that repeated EPDS and MIBS assessments in pediatric practice may help clinicians recognize mothers who experience psychological distress and bonding difficulties and may benefit from closer support. Further prospective studies are required to determine whether such assessments can improve maternal and child outcomes.

## Data Availability

The datasets presented in this article are not readily available because the datasets generated and/or analyzed during the current study are not publicly available because of the risk of participant identification and the need to protect personal anonymity. However, de-identified data and analysis codes may be made available to qualified researchers upon reasonable request from the corresponding author following approval from the institutional Research Ethics Committee. Requests to access the datasets should be directed to TF, tfuji@u-fukui.ac.jp.
