# Supplementary material for: Maternal bonding difficulties and postpartum depression in pediatric clinical practice: a longitudinal study in a Japanese cohort
Source: Front Psychiatry. 2026 Sep 28;17:1939326. doi: 10.3389/fpsyt.2026.1939326 (PMC13655551; doi:10.3389/fpsyt.2026.1939326)
Supplement: Supplementary file 1 [file Table1.docx]

**Supplementary Table S1.** Model selection indices for the Gaussian mixture clustering of LGM-derived factor scores.

| Outcome | Components | Covariance model | BIC | ICL | n per subgroup | Mean classification uncertainty |
| --- | --- | --- | --- | --- | --- | --- |
| MIBS | 1 | XXX | -676.077 | -676.077 | 77 | 0 |
| MIBS | 2 | VVV | -546.506 | -551.912 | 50, 27 | 0.032 |
| MIBS | 3 | VVV | -544.559 | -553.267 | 41, 22, 14 | 0.053 |
| MIBS | 4 | VVV | -545.579 | -554.785 | 41, 9, 13, 14 | 0.057 |
| EPDS | 1 | XXX | -4498.13 | -4498.13 | 512 | 0 |
| EPDS | 2 | VVV | -4011.012 | -4103.465 | 200, 312 | 0.077 |
| EPDS | 3 | VVV | -3874.499 | -3984.026 | 138, 110, 264 | 0.093 |
| EPDS | 4 | VVV | -3751.202 | -3890.019 | 171, 94, 86, 161 | 0.116 |
| EPDS | 5 | VVV | -3843.89 | -4060.26 | 117, 50, 96, 135, 114 | 0.176 |
| EPDS | 6 | VVV | -3751.561 | -3942.554 | 121, 77, 87, 86, 59, 82 | 0.154 |
